# Cauda Equina Syndrome in Neurosarcoidosis

**DOI:** 10.7759/cureus.10069

**Published:** 2020-08-27

**Authors:** Karan Topiwala, Subhendu Rath, Annie Daniel, Avinash Prasad

**Affiliations:** 1 Neurology, Hartford Hospital, Hartford, USA; 2 Neurology, University of Michigan School of Medicine, Ann Arbor, USA; 3 Neurology, University of Connecticut School of Medicine, Hartford Hospital, Hartford, USA

**Keywords:** neurosarcoidosis, cauda equina, immunosuppressive therapy

## Abstract

Neurosarcoidosis (NS) is a mimicker of many infectious, neoplastic, and inflammatory diseases. It most commonly involves the cranial nerves followed by meninges, ventricles, hypothalamic-pituitary axis, spinal cord, and brainstem/cerebellum. While NS myelopathy has been increasingly recognized, pathophysiological/prognostic and management principles in NS-mediated cauda equina (CE) and conus medullaris (CM) syndromes, which constitute a small and rare minority of this subset, remain elusive. We present the case of a 49 -year-old Hispanic man who developed a peripheral facial palsy and primary hypogonadism within a span of 12 months and eventually got diagnosed with NS after he presented with CE syndrome. We also performed an extensive literature review, with a discussion on the underlying pathophysiology and current management recommendations for NS-mediated CE/CM syndrome. CE/CM syndromes in a middle-aged man should prompt the consideration of NS as a possible differential diagnosis. While steroid responsive, the majority of NS-CE/CM patients are left with residual neurodeficits with quick relapses when steroids are tapered, making the case for early institution of immunosuppressive therapies.

## Introduction

Sarcoidosis is an autoimmune multi-organ inflammatory disorder histologically characterized by diffuse infiltration of non-caseating granulomas, with an estimated incidence of 7.6-8.4/100, 000 (2010-2013) in the United States [1]. More than 90% cases present in the third or fourth decade of life with pulmonary involvement, skin or ocular disease, or a combination of these [1]. Nearly 25% patients have involvement of the central nervous system (CNS), but only around 10% actually become symptomatic. Spinal cord involvement is reported to occur in 1-18% of all neurosarcoidosis (NS) cases [1]. Involvement of the cauda equina and/or the conus medullaris (CE/CM) is rare, with only a handful of cases reported worldwide. There are no current guidelines on the management of patients with CE/CM syndrome secondary to NS, with the current standard of care involving a variable taper of corticosteroids with or without long-term immunosuppression (IS) [2]. We present the case of a young man with NS -related CE/CM syndrome (NS-CE/CM) who was treated with steroids with recurrent relapses until IS was initiated. We searched PubMed from database inception to April 2019 with the search terms “[cauda equina] OR [conus medullaris] AND [sarcoidosis] or [neurosarcoidosis]” to review the current literature on the management and prognosis of NS-CE/CM.

## Case presentation

**Figure 1:**
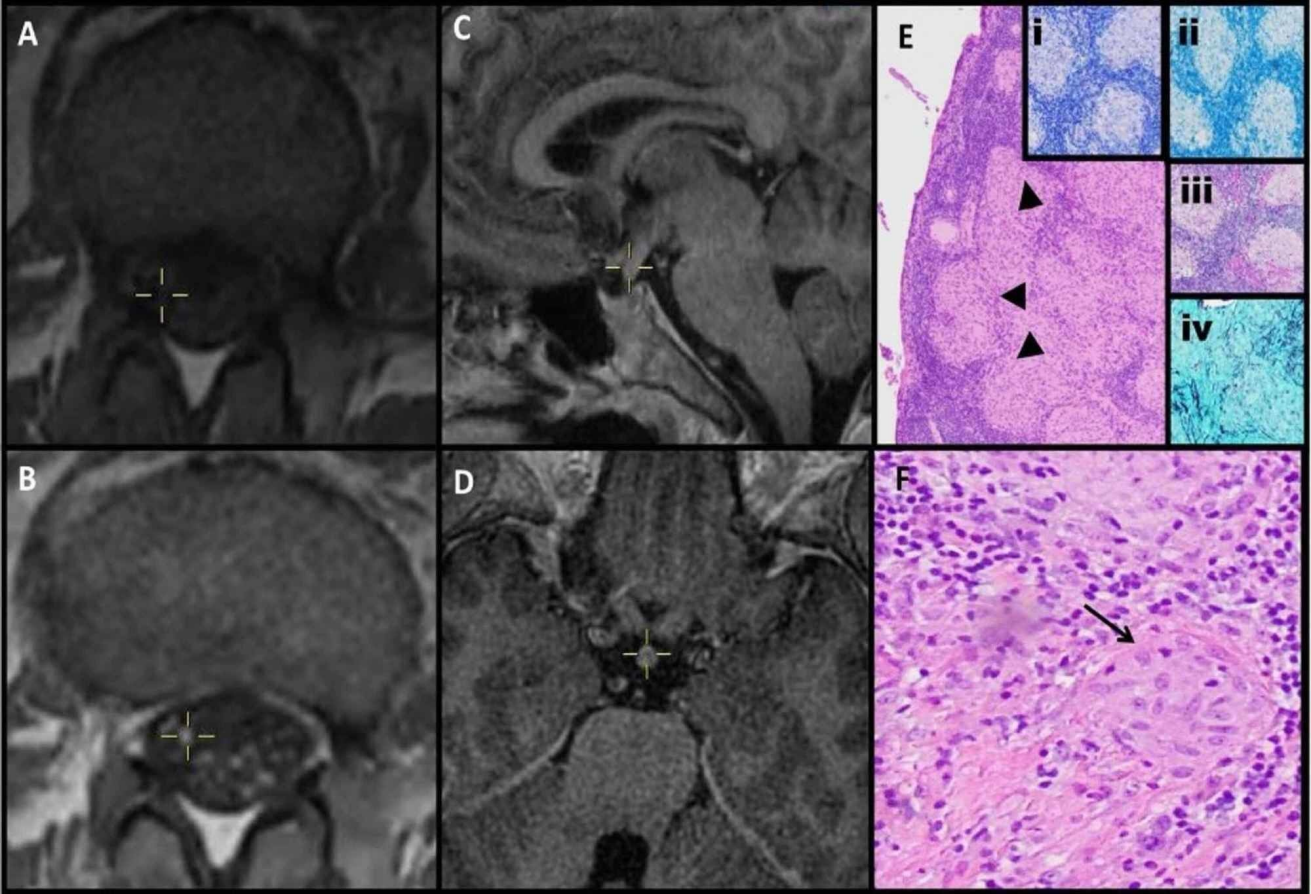
MRI of the lumbar spine showing axial sections through the cauda equina. (A) Pre- and (B) post-contrast images show diffuse nodular enhancement. MRI brain volume imaging (BRAVO) sagittal (C) and axial (D) sections show diffuse enlargement with mild enhancement of the pituitary stalk. (E) Hematoxylin and eosin stain of hilar lymph nodes shows diffuse infiltration of non-caseating granulomas (arrowhead) of the epithelioid cells and multinucleated giant cells (arrow). Special staining with Ziehl-Neelsen (i) and Fite (ii) for mycobacteria, and periodic acid–Schiff (iii) and Gömöri methenamine (iv) for fungal elements was unremarkable.

**Figure 2:**
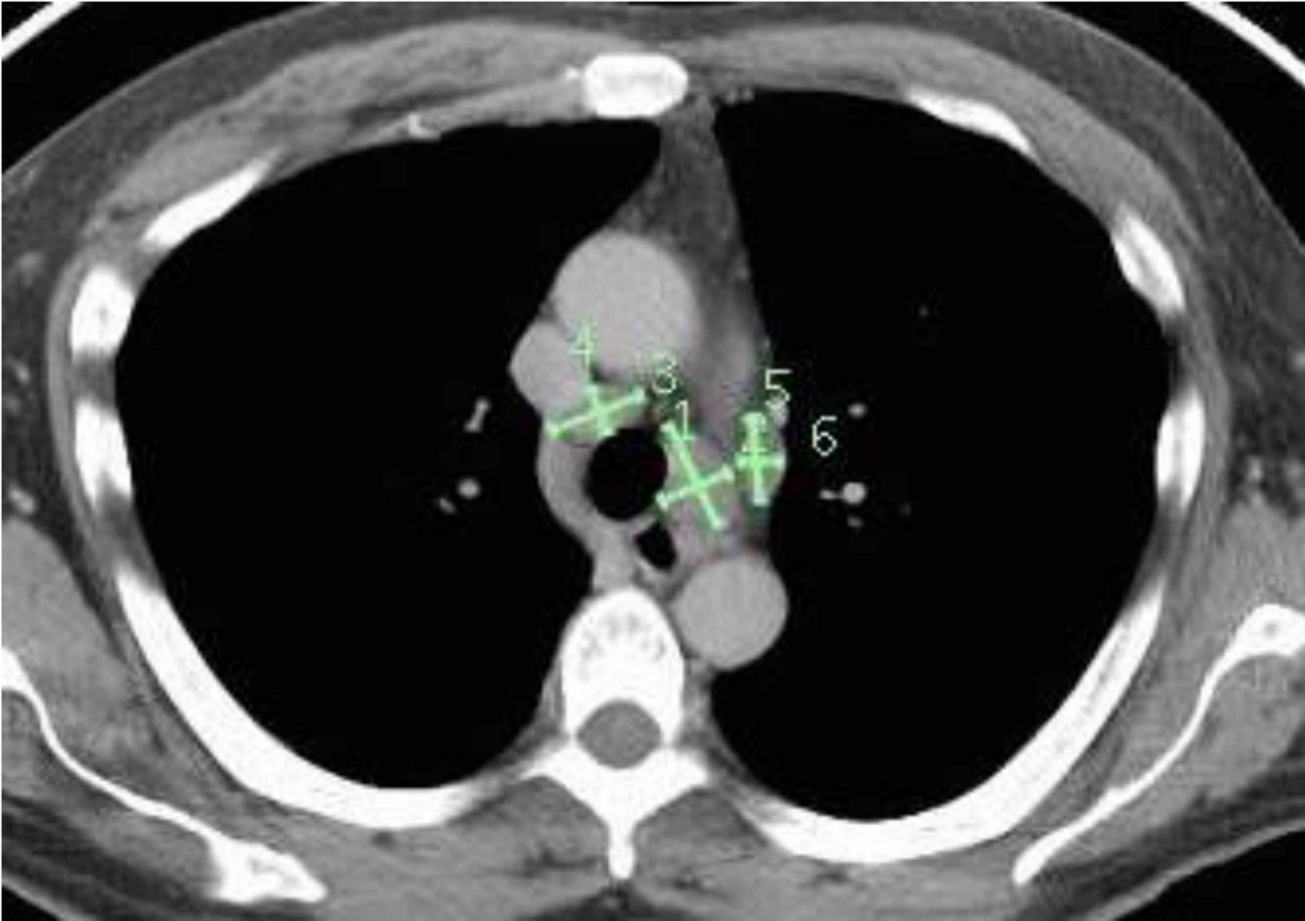
CT of the chest showing extensive mediastinal lymphadenopathy.

A 49-year-old-Hispanic man with a 12-month history of Bell’s palsy and 6-month history of hypogonadotropic-hypogonadism presented to the emergency room with four weeks of sudden-onset, progressive bilateral leg parasthesias and weakness. Neurological exam showed bilaterally asymmetric leg weakness (distal > proximal) with loss of deep tendon reflexes in both legs, except for the right patellar reflex; bilateral stocking neuropathy for large and small fiber modalities with a positive Romberg’s sign; and a wide-based, high-steppage gait. The admission Expanded Disability Status Scale (EDSS) score was 7. Hypogonadism was diagnosed following a six-month history of unintentional weight loss of 40 pounds, dry cough, and low-grade fevers. Magnetic resonance imaging (MRI) brain with a pituitary-protocol completed six weeks prior to admission was normal. A complete blood count along with liver and kidney function tests on admission were unremarkable. Cerebrospinal fluid (CSF) analysis revealed that nucleated cells were 92/cubic (cu) mm (98% were lymphocytes), red blood cells were 9/cu mm, total protein count was 131 mg/dL, and glucose was 45 mg/dL. CSF gram stain did not show any neutrophils or bacteria, and cultures did not demonstrate any growth. Similarly, Ziehl-Neelsen staining did not reveal any acid-fast bacilli, and mycobacterial cultures remained negative. CSF tests for syphilis, herpes simplex virus I/II, cytomegalovirus, and enterovirus through polymerase chain reaction were negative. West Nile virus immunoglobulin (Ig) M/IgG antibodies and CSF cryptococcal antigen screen were negative as well. CSF cytology and flow cytometry were unremarkable. CSF oligoclonal bands were concordant with serum bands. CSF and serum angiotensin-converting enzyme (ACE) levels were within the normal range. CSF and serum paraneoplastic panels were unrevealing. Electromyography (EMG) and nerve conduction velocity (NCV) studies showed a subacute sensory-motor polyneuropathy with axonal and demyelinating features. MRI of the lumbar spine showed diffuse enhancement of the CE nerve roots (Figures 1A, 1B). MRI brain with pituitary protocol showed new diffuse thickening of the pituitary stalk (4 mm from 1 mm six weeks before admission), with mild enhancement (Figures 1C, 1D). Computed tomography (CT) scan of the chest, abdomen, and pelvis showed enlargement of bilateral mediastinal lymph nodes (Figure 2), which were endoscopically biopsied, revealing non-caseating granulomas (Figures 1E, 1F). He was diagnosed with probable NS according to the 2018 consensus criteria by Stern et al. [2] and started on a steroid taper (0.5 mg/kg for four weeks) with significant improvement. He was able to ambulate without support on discharge at one week, with a six-week EDSS score of 1. Repeat EMG six weeks later showed improvement. However, he became steroid dependent with quick recurrence of symptoms upon discontinuation of steroids. He was subsequently started on methotrexate 20 mg per week, with dramatic improvement in strength. At 24-month follow-up, the patient reported stable symptoms, with no subsequent relapses.

## Discussion

Sarcoidosis is an autoimmune multi-system disorder characterized by the presence of non-caseating epithelioid granulomas on histopathology, with an estimated prevalence of 1 to 40 per 100,000 worldwide [1]. Involvement of the nervous system (central and/or peripheral) was first described in 1909 in three men with uveitis, parotid enlargement, fever, and facial palsy [2]. Although around 25% of sarcoidosis patients have involvement of the CNS, only around 10% actually become symptomatic, with 50% to 70% of those presenting with only neurological symptoms [1]. The remainder will become symptomatic within two years of diagnosis [1]. Spinal cord involvement is reported to occur in 1-18% of all NS cases [2]. In a meta-analysis of 1,088 NS patients, only 31% had evidence of systemic disease at presentation, but this increased to 84% later in the disease course [3]. Spinal cord involvement is reported to occur in 0.43% to 1% of all NS cases; however, the aforementioned meta-analysis found spinal cord disease in upto 18% NS cases. Such lesions could be enhancing and longitudinally extensive, and may even have a relapsing-remitting course [3]. This results in a wide differential diagnosis; however, an enhancing (~96%) intramedullary lesion (~ 81%) with leptomeningeal enhancement (~ 48%) spanning more than three spinal levels (~77%) makes NS more likely [2]. It is suggested that parenchymal lesions result from the spread of leptomeningeal inflammation through the Virchow-Robin spaces, which are particularly large at the base of the brain, accounting for their preferential involvement. The lesions evolve in four phases: linear leptomeningeal enhancement, cord expansion, focal > multiple intra-medullary disease with diminishing enhancement, and eventually atrophy [3]. Additionally, extramedullary-intradural lesions (arachnoiditis, nerve root nodules, and root clumping) and extramedullary-extradural lesions (vertebral-body granulomas and discitis) have also been described.

The most common presenting features of NS are cranial neuropathies in 55% (VII [24%] > II [21%] > V [12%] > VIII [11%]), headache in 32%, sensory symptoms in 29%, and motor weakness in 19% (hemiparesis [9%], paraparesis [11%]) [3]. Neuroendocrine dysfunction (pituitary-hypothalamic inflammation), meningeal disease (acute aseptic meningitis, leptomeningeal-pachymeningeal mass lesions), brain parenchymal lesions (mass effect, encephalitis, or seizures), acute-subacute hydrocephalus (communicating or non-communicating) are common presentations [3]. However, CM/CE involvement is extremely rare, with a handful of case series to date.

A diagnosis of NS is typically established via a neural/non-neural biopsy demonstrating the pathologic hallmark of non-caseating epithelioid granulomas in the absence of an infection (tuberculosis, invasive fungal disease), inflammation (inflammatory bowel disease), malignancy (lymphoma), and foreign body/elements (silica) [1]. Consensus diagnostic criteria for NS were only recently published in June 2018 by the Neurosarcoidosis Consortium Consensus Group (10 neurologists and 4 pulmonologists) and endorsed by the World Association of Sarcoidosis and Other Granulomatous Disorders [2]. They require a presentation consistent with NS, both clinically as well as on investigational studies (MRI, CSF, and/or EMG/NCS), and a rigorous exclusion of other differential diagnoses. Thereafter, based on histopathological evidence, they were classified as possible (no pathological confirmation), probable (positive non-neural pathology), and definite (positive neural pathology [type A] or neural plus non-neural pathology [type B]) [2]. Probable and definite were considered to have NS. Thus, a biopsy is always required except in patients presenting with Löfgren’s syndrome (arthritis, erythema nodosum, and bilateral hilar adenopathy, which are present in 9-34% of patients) [1]. Multiple CSF abnormalities have been described (lymphomonocytic pleocytosis [~57%], elevated protein [~60%], low glucose, elevated IgG index, and oligoclonal bands [~20%]) and are most likely in patients with leptomeningeal enhancement [1]. However, no CSF finding is considered pathognomonic of NS, with the CSF being completely normal in upto 30% cases [1]. Biomarkers (including ACE, lysozyme, C-reactive protein, and soluble interleukin-2 receptor assays) in either CSF or serum have not been found to be sensitive nor specific and are not useful in making a diagnosis of NS [3]. Similar to our patient, chest imaging will be abnormal in upto 90% patients [1]. Such imaging may include a chest X-ray, high-resolution chest CT scan or gallium citrate [Ga67] scintigraphy, or, preferably, fluorodeoxyglucose positron emission tomography (PET) scan with the aim of identifying a possible biopsy site [3].

Literature review

Most patients with systemic sarcoidosis do not require therapy [1]. However, currently, NS is primarily treated with systemic corticosteroids, and if it fails, an immunosuppressant is added [4]. The aggressiveness of initial treatment in NS has long been debated; however, in the absence of randomized controlled trials, open-labeled studies are the best available evidence. Scott et al. treated 43 patients with probable or definite NS and followed them for a mean (± SD) of 44.1 months (±43.6) and found that of those treated with an IS (since or within six months of diagnosis) had higher rates of clinical improvement (69% vs. 35%) [4]. Joubert et al. followed 234 NS patients (one of the largest NS cohort studies available) for >60 months (in 68.4% cases) and found a 28% relapse-free survival rate with the use of an IS [4]. The 10-year survival rate was 89%, with older age (HR: 1.64), peripheral nervous system disease (HR: 6.75), and higher baseline EDSS score (HR per point: 1.21) being associated with higher mortality. Despite good survival rates, the 10-year relapse rate was high (86.2%) with encephalic disease [4]. Spinal NS is considered to be relatively refractory to corticosteroids, with one 29-patient series reporting an IS in 83% cases [4]. Optimal management of NS when presenting as CE/CM syndromes remains poorly studied, with variable response to corticosteroids. Contradictory to prior observations, in the study by Joubert et al., myelopathy was not associated with poorer outcomes. Monoclonal antibodies such as infliximab have also been used in patients with chronic steroid-resistant pulmonary and NS with significant clinical and radiographic improvement [3]. A review of NS-CE/CM cases published to date (Table 1) revealed that 68% (n=17) were men with a median age of 40.94 years (SE: 13.78). Of the patients, 35% (n=7/20) and 16% (n=3/19) had a history of prior systemic disease and neurological symptoms, respectively. The median duration of symptoms prior to presentation was 18 months (SE: 7.84). Biopsy was performed on neural tissue in 50% (n=11/22), non-neural tissue in 36% (n=8/22), and both in 14% (n=3/22). Of the 22 patients, 68% (n=152) were treated with steroids only, with 23% (n=5) receiving an IS medication. The overall recovery was reported as moderate or poor in 87% (n=20/23), while only 13% (n=3/23) were asymptomatic at the last follow-up. Further research in the field is needed with multi-center randomized controlled trials assessing the role of simultaneous initiation of steroids along with long-term immunosuppressive agents in patients with NS-CE/CM syndrome.

**Table 1:** Review of published case reports of NS presenting with CE/CM syndromes AA, African-American; ACE, angiotensin-converting enzyme; ACTH, adrenocorticotrophic hormone; AFO, ankle-foot orthotics; C, Caucasian; CE, cauda equina; CM, conus medullaris; CSF, cerebrospinal fluid; D, day; EMG/NCV, electromyography and nerve-conduction-velocity; SR, significant response; MR, moderate response; LAD, lymphadenopathy; PR, poor response; P, prednisone; MTX, methotrexate; N, neural; NN, non-neural; NS, neurosarcoidosis; -, unavailable; B/l, bilateral Note: conus involvement was either radiographic or defined as loss of ankle reflex along with an asymmetric loss of knee reflex, bowel or bladder dysfunction, erectile dysfunction, and saddle anesthesia.

No.	Study	No. of cases	Age/ Sex	Race	Prior neurological symptoms	Clinical prodrome (months)	Systemic disease	Mediastinal LAD	Leptomeningeal enhancement	Conus involvement*	EMG/NCV	CSF/serum ACE	Diagnostic biopsy	Treatment	Last follow-up	Response to steroids
1.	Wiederholt and Siekert [5]	1	30/F	-	-	72	-	-	-	-	-	-/-	N (meninges)	Spinal radiation, chloramphenicol, ACTH, steroids	15 yrs.	MR (residual neurodeficits)
2.	Emery et al. [6]	1	35/M	-	-	24	-	-	-	-	-	-/-	NN ( hilar lymph nodes)	P	5 mo.	MR (residual neurodeficits)
3.	Campbell et al. [7]	1	40/M	-	Yes	18	No	-	No	Yes	Denervation with fasciculations in both quadriceps, NCV were normal	- / -	N (cauda equina)	P (40 mg/d) x 10 d	18 mo.	MR (walk without assistance, no/resolved sphincter dysfunction)
4.	Cooper at al. [8]	1	22/M	-	-	3	Yes	-	-	-	-	- / -	-	Steroids	-	-
5.	Baron et al. [9]	1	18/F	-	No	2	Yes	-	-	No	-	- / -	N (cauda equina)	P (80 mg/d)	-	-
6.	Zajicek [10]	2	48/M	-	-	4	-	-	-	-	-	-/-	N (meninges)	Steroids, anti-tubercular treatment	5 yrs.	PR (worse, with additional neurodeficits)
7.			25/F	-	-	4	-	-	-	-	-	-/-	N (meninges)	P, azathioprine	3 mo.	MR (steroid dependent)
8.	Chamaillard et al. [11]	1	69/M	-	-	180	-	-	-	-	-	-/-	NN (salivary gland)	Steroids	4 mo.	MR (residual neurodeficits)
9.	Ku et al. [12]	1	52/F	AA	No	6	No	Yes	Yes	Yes	Denervation of both tibialis anterior, gastrocnemius, and L4-S1 paraspinal musculature, NCV were normal	- / normal	N (cauda equina) + NN (hilar LN)	P (50 mg/d) x 1 mo.; "low dose" x 7 yrs.	7 yrs.	PR (need AFO/cane at 7-year follow-up); 1st reported case of CM disease
10.	Weissman et al. [13]	1	37/M	C	No	4	No	Yes	-	Yes	-	- / -	N (L1-sacral epidural mass) + NN (hilar LN)	P x 4 mo.	7 mo.	SR (asymptomatic)
11.	Jallo et al. [14]	1	44/M	C	No	0.5	No	No	Yes	Yes	-	- / normal	N (cauda equina)	IV dexamethasone - oral P ("prolonged" duration)	DC	MR (ambulate without assistance)
12.	Abrey et al. [15]	2	62/F	AA	No	3	No	Yes	Yes	Yes	B/l L5-S1 radiculopathy	Normal/normal	N (lumbosacral meninges) + NN (hilar LN)	-	5 yrs.	PR = B/l AFO + crutches
			45/M	C	No	24	No	-	Yes	Yes	-	Normal/normal	-	"steroids"	18 mo.	PR (persistent sensory and gait changes, impotence)
13.	Bassam et al. [16]	1	22/M	-	No	3	Yes	Yes	Yes	Yes	-	- / -	NN (supraclavicular lymph nodes)	P (60 mg/d) x 2 mo.	2 mo.	SR (asymptomatic)
14	Koffman et al. [17]	3	36/M	AA	No	2	No	Yes	-	Yes	Denervation of L2-L3 levels to T4 levels	- / -	NN (hilar lymph nodes)	P (60 mg/d); dependent on P 10 mg/day	U	MR (residual neurodeficit)
			30/M	AA	No	6	No	No	Yes	Yes	-	- / -	N (cauda equina)	P (60 mg/d); dependent on P	U	MR (Ambulates with cane)
			38/F	AA	Yes	8	Yes	Yes	-	Yes	Denervation of left tibialis anterior, gastrocnemius, and lumbar paraspinal; NCV were normal	normal / -	NN (hilar lymph nodes)	P (60 mg/d); P 40 mg/d + azathioprine 200 mg/d	1 yr.	MR (residual and new neurodeficits)
15.	Verma et al. [1]	1	51/M	-	No	6	Yes	No	Yes	Yes	L5-S1 radiculopathy, denervation. NCV normal	- / -	N (lumbosacral meninges)	P (80 mg/d) x 6 mo.; MTX (25 mg/wk.) x 3 mo.	2 yrs.	SR (asymptomatic)
16.	Bode et al. [18]	3	49/F	-	Yes	2	No	No	Yes	Yes	-	Normal/normal	N (cauda equina)	P (60 mg/d)	17 mo.	PR (permanent vision loss) + SR (CE/CM lesions were asymptomatic)
			69/M	-	No	10	No	No	Yes	Yes	-	Normal/normal	N (cauda equina)	P	6 wks.	MR (residual neurodeficit)
			38/M	AA	No	0.75	No	No	Yes	Yes	Denervation of B/L LE muscles with polyradiculopathy; Delayed conduction velocities	Normal/normal	NN (lacrimal gland)	P (60 mg/d); P 5 mg/d x indefinitely	8 yrs.	MR (weak foot eversion/inversion B/l)
17.	Prelog et al. [19]	1	24/M	-	No	48	Yes	Yes	Yes	Yes	-	-/-	-		5 yrs.	PR (bladder, sexual dysfunction)
18.	Kaiboriboon et al. [20]	1	58/F	-	No	0.75	Yes	No	No	Yes	Acute denervation in left lower extremity	Normal / -	NN (lymph nodes)	P (60 mg/d); MTX	2 yrs.	MR (ambulate without assistance)

## Conclusions

Involvement of the spinal cord in NS is rare. The current body of evidence on the management and prognosis of patients with NS-CE/CM syndrome is based on short case series and suggests that while the disease is steroid responsive, the vast majority of these patients will be left with residual neurological deficits with quick relapses when steroids are tapered. Prospective studies evaluating the role of IS at initial presentation of spinal cord disease in patients with NS are urgently required to minimize the morbidity caused by this disorder.
